# Prandial States Modify the Reactivity of the Gustatory Cortex Using Gustatory Evoked Potentials in Humans

**DOI:** 10.3389/fnins.2015.00490

**Published:** 2016-01-05

**Authors:** Agnès Jacquin-Piques, Stéphanie Gaudillat, Thomas Mouillot, Vincent Gigot, Sophie Meillon, Corinne Leloup, Luc Penicaud, Laurent Brondel

**Affiliations:** ^1^Centre des Sciences du Goût et de l'Alimentation, UMR 6265, Centre National de la Recherche Scientifique, Institut National de la Recherche Agronomique, University of Bourgogne Franche-ComtéDijon, France; ^2^Department of Clinical Neurophysiology, University HospitalDijon, France; ^3^Department of Hepato-gastro-enterology, University HospitalDijon, France

**Keywords:** gustatory evoked potentials, high time resolution, food intake, pleasantness, primary taste cortex, gut hormones

## Abstract

Previous functional Magnetic Resonance Imaging studies evaluated the role of satiety on cortical taste area activity and highlighted decreased activation in the orbito-frontal cortex when food was eaten until satiation. The modulation of orbito-frontal neurons (secondary taste area) by *ad libitum* food intake has been associated with the pleasantness of the food's flavor. The insula and frontal operculum (primary taste area) are also involved in reward processing. The aim was to compare human gustatory evoked potentials (GEP) recorded in the primary and secondary gustatory cortices in a fasted state with those after food intake. Fifteen healthy volunteers were enrolled in this observational study. In each of two sessions, two GEP recordings were performed (at 11:00 am and 1:30 pm) in response to sucrose gustatory stimulation, and a sucrose-gustatory threshold was determined. During one session, a standard lunch was provided between the two GEP recordings. During the other session, subjects had nothing to eat. Hunger sensation, wanting, liking, and the perception of the solution's intensity were evaluated with visual analog scales. GEP latencies measured in the Pz (*p* < 0.001), Cz (*p* < 0.01), Fz (*p* < 0.001) recordings (primary taste area) were longer after lunch than in the pre-prandial condition. Fp1 and Fp2 latencies (secondary taste area) tended to be longer after lunch, but the difference was not significant. No difference was observed for the sucrose-gustatory threshold regardless of the session and time. Modifications in the primary taste area activity during the post-prandial period occurred regardless of the nature of the food eaten and could represent the activity of the frontal operculum and insula, which was recently shown to be modulated by gut signals (GLP-1, CCK, ghrelin, or insulin) through vagal afferent neurons or metabolic changes of the internal milieu after nutrient absorption. This trial was registered at clinicalstrials.gov as NCT02472444.

## Introduction

Neuronal recording using electrophysiology in rodents and primates and functional neuroimaging in humans have been conducted to assess how food intake modulates brain activation. Earlier studies showed decreased activation following the consumption of food eaten to satiation (Rolls, 2005, 2006, 2007, 2008, 2011, 2012, 2015). The modification of the cortical activity after food intake was found in the orbito-frontal cortex (Critchley and Rolls, 1996) and was related to sensory-specific satiety (Rolls, 2005, 2006, 2007, 2008, 2011, 2012, 2015) since cortical activity was not changed when primates or humans were stimulated by other non-consumed foods. Studies have also recently suggested that neuronal activation of the orbito-frontal cortex correlated with either liking or wanting ratings, suggesting that this area of the cortex plays a role in the reward processing pathway (Jezzini et al., 2013; Jiang et al., 2015; Rolls, 2015) and correlates with the subjective pleasantness of taste. Hence, previous data provide evidence that the pleasantness of a food's flavor is represented in the orbito-frontal, cingulate, and medial prefrontal cortex (Rolls, 2008; Jezzini et al., 2013), which corresponds to the secondary gustatory area.

In a different way, it has been observed that responses in the primary gustatory cortex (insula and opercular cortex) correlated with the subjective intensity, temperature, viscosity, and fat texture of foods (Kringelbach et al., 2003; Small et al., 2003; Rolls, 2004, 2007, 2008, 2012; Sewards, 2004; Ohla et al., 2012b). However, recent studies have noted that the insula and frontal operculum, also participate in modulating the hedonic value of taste (Small et al., 2003; Menon and Uddin, 2010; Frank et al., 2013; Jezzini et al., 2013). In fact, electrophysiological studies in rats showed that taste palatability was coded in both the primary gustatory cortex and the medial prefrontal cortex (Jezzini et al., 2013). Moreover, functional Magnetic Resonance Imaging (fMRI) in humans showed that the left dorsal anterior insula and the opercular region responded to unpleasant tastes (Small et al., 2003).

fMRI was used to study about the role of food intake and the prandial state in taste area activation in humans (Rolls, 2011, 2012, 2015). Despite its good spatial resolution, its time resolution is lower than that achieved with electroencephalography, especially with evoked potentials. High time resolution is required to obtain reliable measurements of latency of activity in the taste cortex. Electroencephalography and evoked potentials have the advantage of millisecond time resolution (Ohla et al., 2012a; Gemousakakis et al., 2013). Recording gustatory evoked potentials (GEP) on the scalp is a safe and non-invasive method to study gustatory cortex activity (Ohla et al., 2012a). GEP latency, amplitude, and duration relative to taste receptor stimulation provide a more precise description of activity of the taste cortex than fMRI. To our knowledge, no study has been conducted using GEP to assess the influence of the prandial state in gustatory area activity.

As it has been reported that the activity of both the primary and secondary gustatory cortices correlates with the subjective pleasantness of taste and as it is well-known that hedonic sensations for foods depend on the prandial state, we hypothesized that food intake could modulate cortical activity in both the primary and secondary taste areas. To test this hypothesis, we compared GEP recording, which is a high time resolution technique, in the primary and secondary gustatory cortices in young healthy subjects after sucrose stimulation in two physiological situations: fasting vs. food intake. The postulate was that modulating the prandial state should manifest as lengthening GEP latencies and decreasing GEP amplitudes.

## Methods

### Subjects

Fifteen healthy volunteers (seven men, eight women) were enrolled in this observational study. The mean age was 28 ± 7 years old (range: 22–35 years old), and the mean BMI was 22 ± 3 kg.m^−2^ (range: 19–25 kg.m^−2^). There were no significant gender differences for these parameters. All were non-smokers, and none had dental or neurological problems, or any remarkable medical history. We excluded subjects under current medical treatment, or who were overweight (BMI > 25 kg/m^2^) or underweight (BMI < 19 kg/m^2^).

The subjects agreed to participate with written consent after being informed about the nature and aims of the experiments. The study was approved by the regional Ethics Committee of Burgundy, France, in accordance with the latest revision of the Declaration of Helsinki and European Law (ISO EN 14155). This trial was registered at clinicalstrials.gov as NCT02472444.

### Experimental design

**Figure 1:**
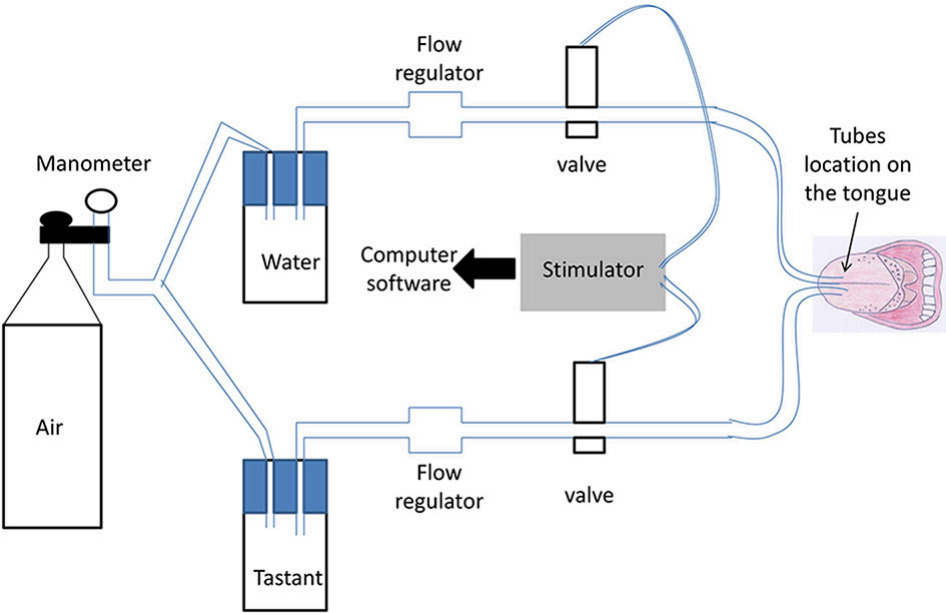
**Schematic view of the taste delivery system**. Water and taste solutions were driven through the system by compressed air (controlled through a manometer). Two parallel silicone tubes were used; one for water and the other for the taste solution. Switching between water and the taste solutions was performed by two electromagnetic valves controlled by an electronic device. Each subject put the two parallel tubes on the middle of his/her tongue in his/her mouth. Solutions were delivered to the tongue through a hole at the end of each tube.

The taste delivery system is described in Figure 1. Water and taste solutions were driven through the system by compressed air (controlled through a manometer). Two parallel silicone tubes were used: one for water and the other for a taste solution. Water and taste solutions were switched from one to the other by two electromagnetic valves controlled by an electronic device. This electronic device (stimulator) sent a signal to computer software (SystemPLUS EVOLUTION, 2007 Micromed S.p.A, Italy) when a taste solution was administered (with 1 ms precision), to obtain a precise time recording of GEP.

Each subject put the two parallel tubes on the middle of his/her tongue in his/her mouth. The extremity of the semi-rigid tubes (silicone tubing, P/N 10025-02S, Bio-Chem valve) was placed at 1.5 ± 0.5 cm from the dental arch on the midline of the tongue (same distance for each subject). Due to their rigidity, the tubes could not deviate from this position. Solutions were delivered to the tongue through a hole at the extremity of each tube. A taste solution was intermittently delivered through the first tube (flow rate = 200 mL/h). During the period without the taste solution, water was continuously delivered through the second tube (flow = 100 mL/h) to minimize the likelihood that subjects would feel different sensations between injections from the two tubes. The flow rate of 200 mL/h of taste solution was chosen because it allowed the uniform stimulation of a large lingual surface (21–24 cm^2^, tested just after stimulation of the tongue by a methylene blue solution in a preliminary work). The same oral receptive fields were therefore activated in each subject. The flow rate of 100 mL/h of water was chosen because it did not induce somatosensory differences compared with the flow rate of 200 mL/h, and it meant that participants did not have swallow too often, which could lead to artifacts on the GEP recording. This was tested in a preliminary study and verified in the control recordings (stimulation by water through the two tubes). Air was purged from the taste delivery system to avoid a feeling of air on the tongue.

Each GEP session lasted ~40 min: 20 min to prepare for the GEP recording and 20 min for the GEP recording itself. In each recording session, a stimulus was delivered 20 times for 1 s each time. Each stimulus was separated by a 1-min interval in which water alone was delivered. During each GEP recording, subjects listened to quiet music through headphones to mask the switching clicks of the electromagnetic valves. No evoked potential was recorded in our experiment in response to quiet music (checked with control GEP recordings). The subjects also had to close their eyes to avoid light stimulation. Hence, no blink artifacts contaminated the recordings. Participants were instructed to swallow after receiving the taste stimulus. Control GEP recordings showed that swallowing or mouth movement did not lead to frontal electrode artifacts.

### Tastant stimulation, food intake, and subject's sensations

The tasting stimulus was a solution of sucrose applied in one concentration: 10 g per 100 mL of water (Evian water which is almost deionized; stimulation by Evian water alone did not induce GEP).

**Figure 2:**
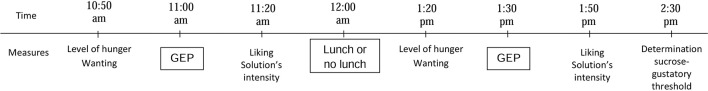
**Design of the experiments**. To measure hunger, wanting, liking ratings, and perceived intensity of the sucrose solution, the subjects had to answer the four following questions: “Are you hungry?,” “Do you feel like eating?,” “How palatable was the sucrose solution?,” and “How intense was the sucrose solution?,” The responses were scored using a 10-cm visual analog scale ranging from “Not at all” to “Extremely.” The same measurements in that order were performed in the two separate sessions (feeding and fasting). (GEP, Gustatory-evoked potentials).

Subjects were investigated in two parallel sessions separated by at least 1 day. Subjects were asked to eat their usual breakfast before 8:00 am and not to eat or drink anything except water until the first GEP recording. During one session, each subject had two GEP recordings, the first at 11:00 am, and the second at 1:30 pm (Figure 2). In the feeding session, lunch was provided to the participants between the two GEP recordings (at 12:00 am) Lunch was composed of grated carrots with lemon juice, beef ravioli with tomato sauce, bread, and cheese. The participants did not eat the same amount of calories because each food was eaten *ad libitum* until satiation. No dessert was served to avoid sweet food before a sucrose GEP recording. The food was eaten in 15–20 min. In the fasting session, no lunch was served between the two GEP recordings. The two sessions were randomly assigned.

Before each GEP recording, subjects were asked to rate their level of hunger and desire to eat (wanting) using a 10-cm visual analog scale (Figure 2). After each GEP recording, subjects were asked to define the hedonic value (liking) and the perceived intensity of the solution using a 10-cm visual analog scale (VAS). They had to answer the four following questions: “Are you hungry?,” “Do you feel like eating?,” “How palatable was the sucrose solution?,” and “How intense was the sucrose solution?.” The VAS responses ranged from “Not at all” to “Extremely.”

### GEP recording and data analysis

**Figure 3:**
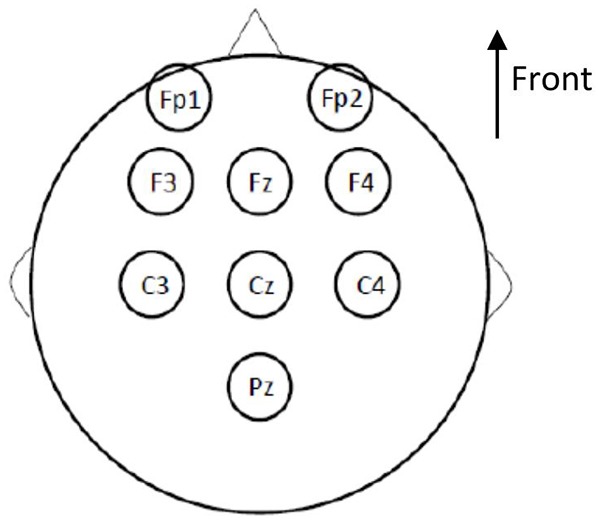
**Cortical sites of gustatory evoked potentials (GEP) recording according to the international 10–20 system (conventional EEG recording)**. Nine sites were taken into account: Pz, Cz, C3, C4, and Fz for measurements of the primary gustatory cortex; and F3, F4, Fp1, and Fp2 for measurements of the secondary gustatory cortex. The electrodes were referenced against linked earlobes. The ground electrode was placed on the forehead.

Electroencephalographic (EEG) signals were recorded according to the international 10–20 system, using a conventional EEG recording. Nine sites were recorded for measurements in the primary gustatory cortex (Pz, Cz, C3, C4, Fz), and the secondary gustatory cortex (F3, F4, Fp1, Fp2; Figure 3). The primary gustatory cortex correspond to the insula and the operculum cortex, and activity was recorded at positions Pz, Cz, C3, C4, and Fz in previous studies (Hummel et al., 2010; Singh et al., 2011). As for other cortical evoked potentials, the midline electrodes, the vertex electrode in particular, were the site of the most pronounced amplitudes of GEPs (Kobal, 1985). The secondary cortical taste area is located in the orbito-frontal and prefrontal cortices, and activity there was recorded by frontal electrodes, especially Fp1 and Fp2, in the international 10–20 system. Electrodes were referenced against linked earlobes (A1 + A2). The ground electrode was placed on the forehead.

EEG measurements were amplified, filtered and digitized using Micromed software (SystemPLUS EVOLUTION, 2007 Micromed S.p.A, Italy) with the following data: time constant 1 s, sampling frequency 2048 Hz, 200 Hz low-pass filter, 0.4 Hz high-pass filter, 50 Hz filter. Recordings were additionally filtered offline. GEPs were averaged after the recording for each session (average of 20 stimuli; Hummel et al., 2010; Singh et al., 2011).

GEP analyses were performed using the same software. Initial latency (ms), amplitude (μV), and duration (ms) of the GEPs were noted for each recorded electrode. The initial latency was defined as the time between stimulus delivery and the onset of the increase in potential. The amplitude of each response was calculated from positive to negative peaks. The duration of the GEPs was calculated between the end and the beginning (corresponding to the initial latency) of the GEP. The positive peak corresponded to the peak pointing down whereas the negative peak corresponded to the peak pointing up. GEP was defined by three peaks, as described in previous studies (Hummel et al., 2010; Singh et al., 2011): P1 the first positive peak, N1 the higher negative peak and P2 the second positive peak. The software first averaged the GEPs and then detected the peaks. GEP recordings were analyzed by the same well-trained neurophysiologist, blinded to the type of session (fasting or fed), and were processed with a standard and consistent method of EEG analysis for both prandial states.

### Determination of sucrose-gustatory threshold

Thresholds were determined twice for all participants, once at the end of the feeding session, and the other at the end of the fasting session, using a 3-alternative forced-choice procedure (Keast and Roper, 2007; Chevrot et al., 2014; Low et al., 2014), in which participants were provided with successive sets of three samples.

Briefly, each set contained two control samples and one stimulus sample. Within each set, participants had to indicate which sample was different from the other two. Sets were presented in ascending concentrations from 0.0609 to 1.0824 g sucrose per 100 mL of water (Evian water) spaced by 0.25 log units (six solutions in total). The procedure was stopped when the participant correctly identified the stimulus sample at a given concentration three consecutive times. This concentration was called the sucrose-gustatory threshold for the individual participant.

### Statistical analysis

Initial latency, amplitude and duration of GEPs (located in Pz, Cz, Fz, Fp1, and Fp2 according to the 10–20 system) were analyzed using means and standard errors of the mean (SEM). Mean differences in GEP latency, amplitude, and duration between data obtained in the morning and afternoon recordings, the results from the visual analog scales (hunger sensation, wanting, liking, solution intensity) and sucrose-gustatory thresholds were compared between the feeding and fasting sessions. The analyses were performed using a repeated measures ANOVA (with a single factor: feeding or fasting session). A *p*-value below 0.01 was considered statistically significant (Bonferroni correction for five analyses).

SAS 9.2 (SAS Institute Inc, Cary, North Carolina) was used for all analyses.

## Results

### Sucrose-gustatory threshold

The sucrose-gustatory threshold was similar regardless of the prandial states in all of the participants. It ranged from 0.0609 to 0.3423 g/100 mL of water. The median sucrose-gustatory threshold was 0.1925 g/100 mL of water for both the prandial states.

### Hunger sensation, wanting, liking, and perceived intensity of the solution (Figure 4)

The sensation of hunger and wanting significantly increased with fasting (*p* < 0.001) and significantly decreased after food intake (*p* < 0.001).

**Figure 4:**
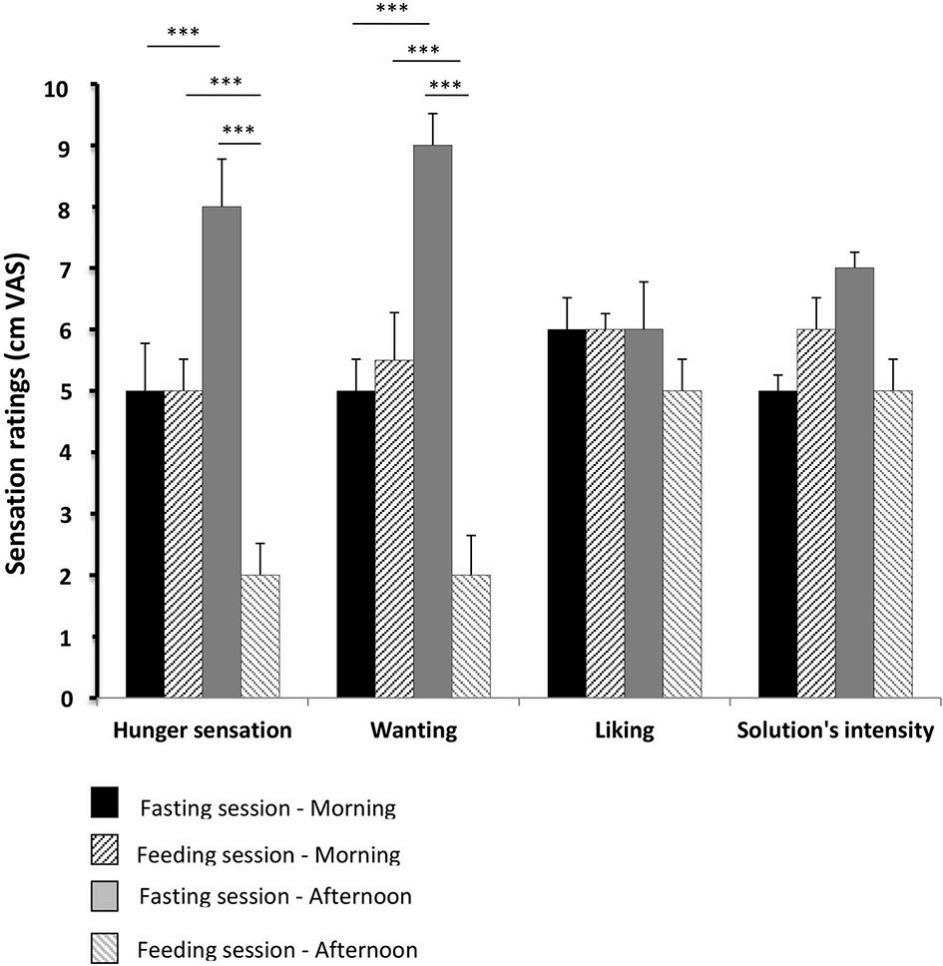
**Hunger sensation, wanting, liking, and perceived intensity of the solution**. These parameters were evaluated using 10-cm visual analog scales (VAS) for the 15 healthy volunteers evaluated in pre- and post-prandial states in both sessions (feeding and fasting sessions, respectively). The results from the visual analog scales (hunger sensation, wanting, liking, solution intensity) were compared between the feeding and fasting sessions, using a repeated measures ANOVA. A *p*-value below 0.01 was considered statistically significant (Bonferronni correction). Results are expressed as the mean ± SEM. ^***^p < 0.001.

Liking and perception of the solution's intensity did not differ significantly from one period or recording session to another.

### GEP parameters

**Figure 5:**
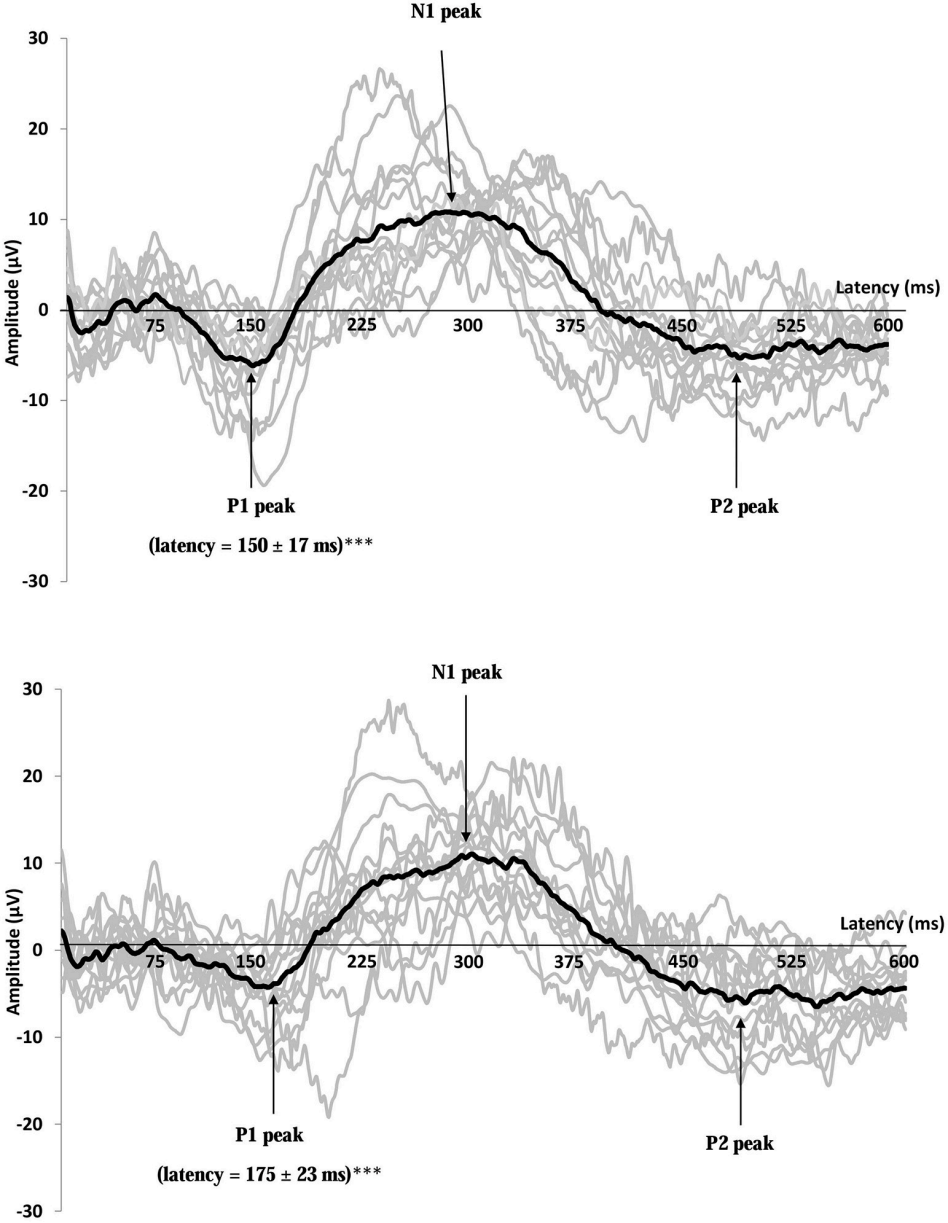
**Recordings of gustatory-evoked potentials (GEP) before (top) and after feeding (bottom) in response to a 10 g/100 mL sucrose solution, on the Cz electrode**. Both figures show the average of GEPs of the 15 participants after stimulation by the sucrose solution (for each subject, the curve corresponds to an average of 20 stimuli). GEP was defined by three peaks: P1 the first positive peak, N1 the higher negative peak and P2 the second positive peak. GEP latency in the primary gustatory cortex was prolonged after food intake ^***^p < 0.001.

**Figure 6:**
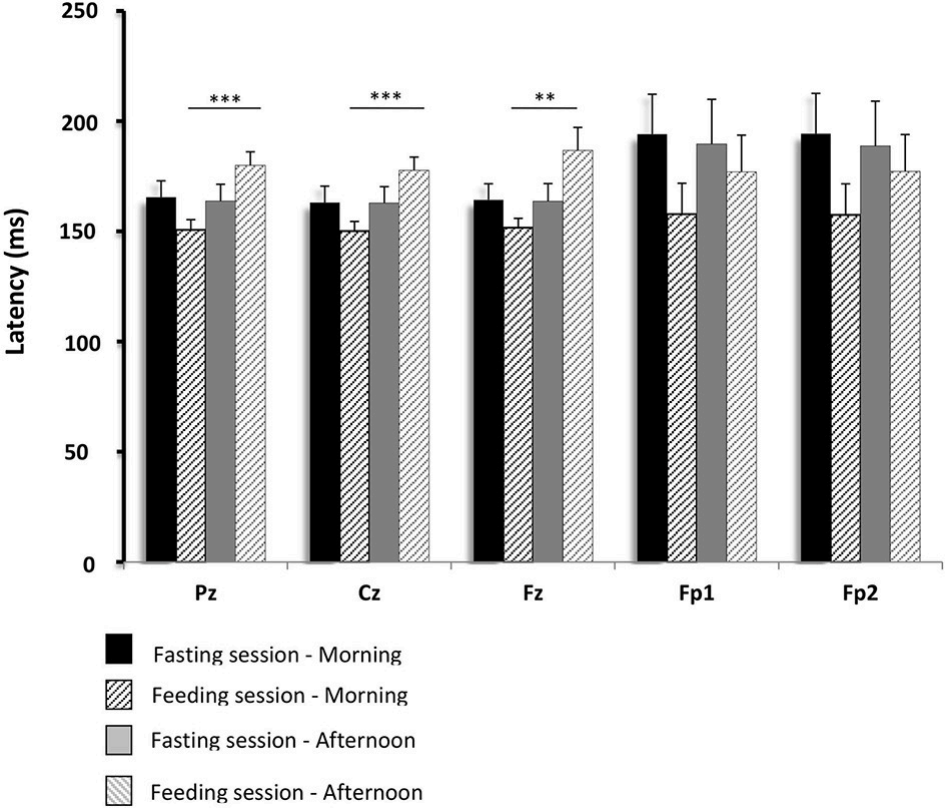
**Comparisons of gustatory-evoked potential (GEP) latencies between the fasting and feeding sessions**. GEPs were recorded in response to sucrose stimulation in the 15 healthy volunteers. Mean differences in GEP latency between data obtained in the morning and afternoon recordings were compared between the feeding and fasting sessions. The analyses were performed using repeated measures ANOVA (with a single factor: feeding or fasting session). A *p*-value below 0.01 was considered statistically significant (Bonferroni correction). Pz, Cz, Fz, Fp1, and Fp2 are the locations of the electrodes on the scalp where GEPs were recorded. Pz, Cz, and Fz correspond to the primary gustatory cortex; Fp1 and Fp2 correspond to the secondary gustatory cortex. Results are expressed as the mean ± SEM. ^**^p < 0.01; ^***^p < 0.001.

**Figure 7:**
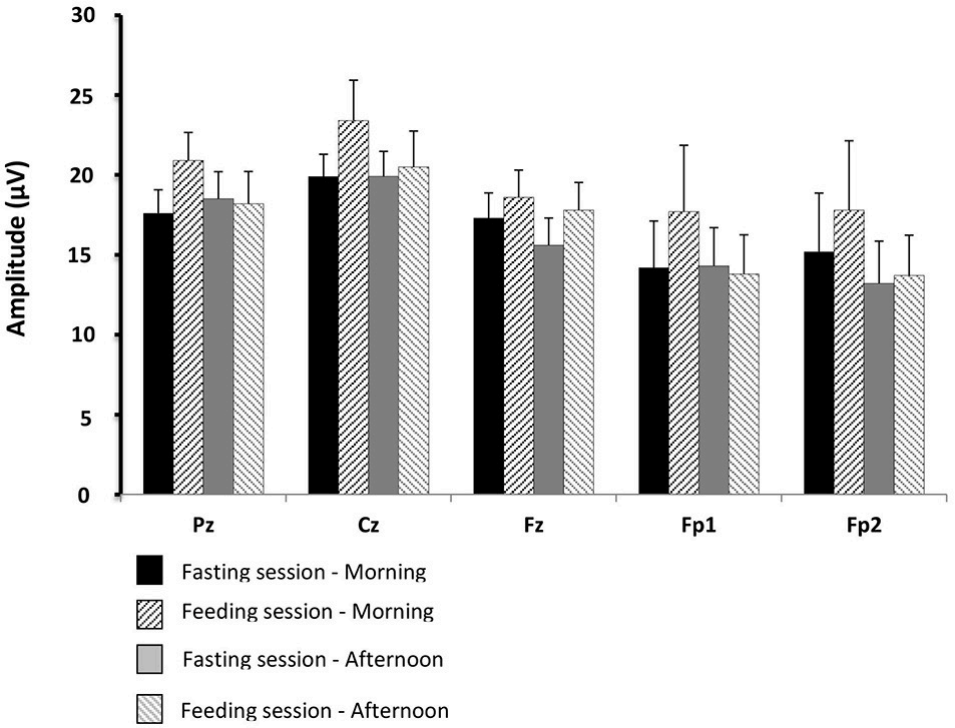
**Comparisons of gustatory-evoked potential (GEP) amplitudes between the fasting and feeding sessions**. For legends, see Figure 6.

**Figure 8:**
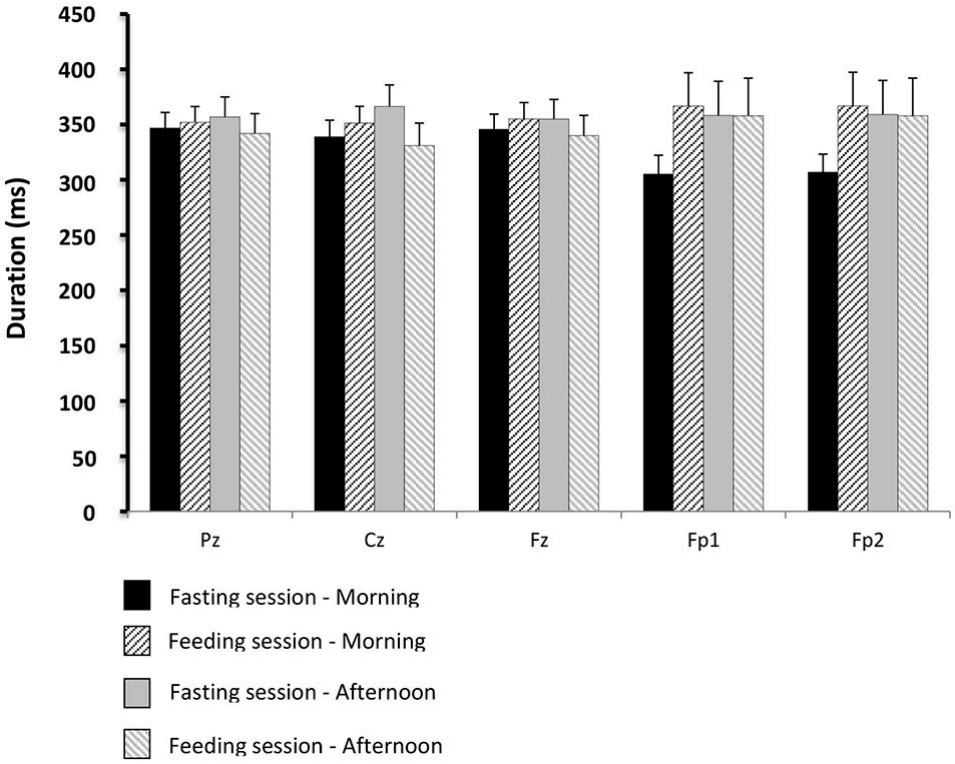
**Comparisons of gustatory-evoked potential (GEP) duration between the fasting and feeding sessions**. For legends, see Figure 6.

The average GEP of all the subjects obtained for the Cz electrodes in the feeding session (before and after lunch) is shown in Figure 5. The values for GEP latency, amplitude, and duration are shown in Figures 6–8, respectively.

There was no statistical difference between male and female participants for GEP parameters.

In the fasting sessions, the GEP latencies were not significantly different between the pre- and post-prandial conditions. However, in the feeding sessions, they were longer after food intake than before. These changes were significant in the Pz (*p* < 0.001), Cz (*p* < 0.001), and Fz (*p* < 0.01) electrodes. No significant change was observed in the secondary gustatory area, although there was a slight trend toward longer latencies in Fp1 and Fp2.

There were no significant differences between the physiological situations (periods of recording and sessions) for GEP amplitude and duration.

## Discussion

This study highlighted the change in the primary gustatory area 1 h after food intake compared with the fasting state: GEP latencies in response to sucrose stimulation lengthened after lunch even though the meal did not contain sweet food. A similar, though non-significant trend, was present in the secondary taste cortex. In contrast, no decrease in GEP amplitudes was noted according to the prandial state.

Some discrepancies should be noted in our findings on cortical changes in the primary gustatory cortex after *ad libitum* food intake when compare with data in the literature. In fact, neurophysiological recordings in monkeys and rodents and functional neuroimaging studies in humans showed decreased activation of the secondary taste cortex. The neuronal cortical activity was found to be modified in the orbitofrontal cortex (Critchley and Rolls, 1996) and not in the primary gustatory area (Kringelbach et al., 2003; Rolls, 2005, 2006, 2007, 2008, 2011, 2012, 2015). In fact, when primates were fed to satiety with glucose, the activity of single neurons in their caudolateral orbito-frontal cortex specifically decreased to zero for the food ingested but continued for foods which had not been eaten, thus reflecting sensory-specific satiety (Rolls, 1987, 1989; Critchley and Rolls, 1996). Neurophysiological recordings in primates thus demonstrate that orbito-frontal neurons respond to foods in a sensory-specific pattern, whereas neurons in the primary taste cortex do not represent the reward value of taste. In fact, in macaque monkeys fed to satiety, the neurons of the insular and frontal opercular primary taste cortex showed no reduction in their firing in response to taste (glucose, for example; Rolls et al., 1988; Yaxley et al., 1988; Rolls, 2015). The same mechanisms exist in rodents. After oral infusion of sucrose until behavioral satiation, positive hedonic reactions were reduced more by oral sucrose than by oral milk (Grill and Norgren, 1978), and the previously eaten food was less frequently eaten (Dwyer, 2005). The sensory-specific satiety mechanism seems to affect only the secondary taste cortex: in rats with lesion of gustatory insular cortices, the sensory-specific pattern is preserved (Balleine and Dickinson, 2000). However, concerning satiety in rats, both responses of the orbito-frontal and the insular cortices were modulated by prandial state (de Araujo et al., 2006).

The main discrepancies noted in our findings on cortical changes when compared with data in the literature could be explained by the presence of two different physiological mechanisms underlying the neuronal changes in the taste cortices. Decreased activation in the orbitofrontal cortex during food intake and its association with decreased pleasantness of the food eaten (Rolls, 2015) may be due to habituation. Habituation results in a specific diminution of response to a stimulus after repetitive confrontations of the organism with it, through a brain mechanism of non-associative learning (McSweeney and Swindell, 1999). In the same way, it has been shown that habituation plays an important role in motivated responses to food in humans (Brondel et al., 2009a). Habituation could explain sensory-specific satiety (Brondel et al., 2009b), which has been reported in several studies (Critchley and Rolls, 1996; Rolls, 2005, 2006, 2007, 2008, 2011, 2012, 2015). In contrast, decreased activation in the primary taste cortex after food intake regardless of the type of food eaten, as in our study, may be due to alliesthesia, which is linked to the internal milieu of subjects (Cabanac, 1999; Brondel and Cabanac, 2007; Jiang et al., 2008). The sequence of physiological events could be as follows: food intake first triggers a sensation of pleasantness, modifies the internal state and gut content. Gut stimulation decreases the pleasantness of food intake and can even lead to displeasure for food by stimulating the gustatory cortex. The lack of food pleasantness is present until the next meal. Two arguments might be put forward to support this hypothesis.

First, it has been reported that the insula and the frontal operculum (parts of the primary gustatory cortex) participate in the perceived pleasantness of taste (Small et al., 2003; Menon and Uddin, 2010; Frank et al., 2013; Jezzini et al., 2013; Huerta et al., 2014), and are involved in the reward processing of food intake (Jiang et al., 2015), as well as other reward pathways, such as food craving-reward and other craving types (smoking, cocaine, drug abuse, etc.) (Huerta et al., 2014). These previous findings may explain why primary gustatory activity was modified in our study 1 h after food intake. Second, the primary gustatory cortex receives multimodal afferent signals, particularly from gustatory and visceral stimulation (Katz et al., 2002; Rolls, 2012; Low et al., 2014). The action of peripheral signals (nutrients, hormones such as ghrelin, leptin, GLP-1, and CCK) on vagal afferent neurons is recognized as an important pathway in regulating food intake (Beglinger and Degen, 2006; Overduin et al., 2012; Pénicaud et al., 2012; Dockray, 2014; Goldstone et al., 2014).

It is difficult to explain more precisely why latencies were lengthened after food intake. The lengthening of evoked potential latencies corresponds to a slowing of neuronal signals from peripheral receptors to cortical areas. Modification of synaptic plasticity in the cortical taste area (Oda et al., 2014) could explain the rapid lengthening of GEP latencies after food intake compared with those recorded in the fasting state 2 h earlier. Other mechanisms implicating changes in presynaptic action potential waveforms could explain modifications of synaptic latency after food intake (Boudkkazi et al., 2011). Differences in GEP latency have been observed between men and women (Hummel et al., 2010), and we have also shown that latencies lengthened with old age (personal unpublished data). These differences are also observed in other types of evoked-potentials (visual or sensory) and certain authors have involved that hormonal changes explain these differences in evoked-potential latencies (Sharma et al., 2015). Obviously, other mechanisms, such as metabolic parameters, cannot be excluded to explain the neuronal reactivity of the primary taste area after food intake.

We also found that the sucrose-gustatory threshold in the fasting state was similar to that in the feeding state. Recent data in the literature suggest that the gustatory threshold is probably influenced by environmental and genetic factors (Low et al., 2014). The absence of variability in the sucrose threshold in our study suggests that the GEP changes in the primary taste cortex were not related to taste receptor desensitization during food intake.

Various technical elements in our experiments could also be pointed out. To record latencies in the taste cortex, we used GEP, a technique with higher time resolution than fMRI (Ohla et al., 2012a; Gemousakakis et al., 2013). The high time-resolution technique is more accurate at detecting changes in GEP latencies in cortical taste area activity. We could also argue that GEP recording in the orbitofrontal cortex is less reliable than that in the primary gustatory cortex because of artifacts due to eye movements and possible activation from an anticipation phenomenon (subjects focused on the taste stimulus; O'Doherty et al., 2002). Moreover, the orbitofrontal cortex receives projections from several cerebral sensory areas (visual, auditive, olfactive, gustative, and somatosensory areas; Rolls, 2004). To counterbalance these interferences, we averaged the GEPs and had the subjects listen to music to mask the noise from the environment, and close their eyes to avoid light stimulation.

Our protocol has several limitations. First, the insula is not easy to record using GEPs because it is deep in the brain. However, the frontal operculum, which plays the same role as the insula in food intake and the reward processing pathway, is easy to record. Second, we cannot totally exclude the possibility that somatosensory cortical responses may have contributed slightly to the GEPs (Cerf-Ducastel et al., 2001; Sewards, 2004), though the high-time resolution technique we used was able to distinguish between gustatory and somatosensory responses (different latencies). We cannot totally exclude the possibility that the gustatory cortex was also activated by water (Small et al., 1999; de Araujo et al., 2003; Rolls, 2004; de Araujo and Simon, 2009). However, the continuous water stimulation in our experiment would have attenuated or canceled the somatosensory response (by habituation of the somatosensory system) while maintaining the gustatory cortical response (Kobayakawa et al., 1996; Ohla et al., 2012a). Third, although uniform stimulation of a large lingual surface was applied to activate the same oral receptive fields in each subject, we cannot exclude small movements of the tube in the mouth. However, no regional differences in suprathreshold intensity were observed for the sweet taste between subjects in another study (Feeney and Hayes, 2014). Fourth, we cannot exclude the possibility of a small delay (a few milliseconds) between the delivery of the taste solution and activation of the taste receptors. However, as we use the same protocol for all the GEP recording sessions, the validity of our results is quite certain. Fifth, it would have been informative to measure metabolic patterns and GEP recordings in response to different concentrations of sucrose solution to know if there was a dose response relationship. However, our study was a pilot study with exploratory results which should be completed by further work. We intended to measure metabolic patterns, such as leptin or ghrelin and test a dose-response in GEP recordings in future experimental studies. Finally, the association of GEP recording and fMRI would bring more information to the study thanks to the good spatial resolution of fMRI. In fact, fMRI could help to give more details about the exact cortical location of neuronal activation. On the other hand, fMRI would be necessary to study other deep cerebral areas, which cannot be explored by EEG and GEP, and which are known to be involved in the sensory-specific satiety and reward pathways. In fact, it has been shown in previous studies that activation of hypothalamus, thalamus, amygdala, hippocampus, and parahippocampus cortices was modified during the physiological states of hunger and satiety (Haase et al., 2009).

In conclusion, our study showed that GEP latencies in response to sucrose stimulation lengthened after food intake, even though the meal did not contain sweet food. Modifications in GEPs in the primary gustatory area (Pz, Cz, Fz electrodes) were significant. This result demonstrates that activity in the primary taste area changes after food intake regardless of the food eaten. These neuronal changes could be due to modifications of the internal state and gut stimulation after food intake and could partly explain the mechanism related to negative alliesthesia. Further studies are needed to determine the mechanisms underlying these modifications.

## Author contributions

AJ, CL, LP, and LB designed the study. AJ, SG, TM, and SM conducted the research. VG provided essential materials necessary for the research. AJ performed statistical analyses. AJ, CL, LP, and LB analyzed data. AJ wrote the paper. AJ had primary responsibility for final content. All the authors reviewed and approved the final manuscript.

## Funding

This work was supported by CNRS, INRA, the University of Bourgogne Franche-Comté, the FEDER and the Dijon Association for Neurosciences (ADSNB), France.

### Conflict of interest statement

The authors declare that the research was conducted in the absence of any commercial or financial relationships that could be construed as a potential conflict of interest.

## Abbreviations

CCK: cholecystokinin
GEPs: gustatory evoked potentials
GLP-1: glucagon-like peptide-1.
